# Detecting spikes of wheat plants using neural networks with Laws texture energy

**DOI:** 10.1186/s13007-017-0231-1

**Published:** 2017-10-13

**Authors:** Li Qiongyan, Jinhai Cai, Bettina Berger, Mamoru Okamoto, Stanley J. Miklavcic

**Affiliations:** 1 School of Engineering, Beijing Forestry University, Beijing, 100083 China; 2 Phenomics and Bioinformatics Research Centre, University of South Australia, Mawson Lakes, SA 5095 Australia; 3 School of Agriculture, Food and Wine, University of Adelaide, Urrbrae, SA 5064 Australia; 4 The Plant Accelerator, Australian Plant Phenomics Facility, University of Adelaide, Waite Campus, Urrbrae, SA 5064 Australia

## Abstract

**Background:**

The spike of a cereal plant is the grain-bearing organ whose physical characteristics are proxy measures of grain yield. The ability to detect and characterise spikes from 2D images of cereal plants, such as wheat, therefore provides vital information on tiller number and yield potential.

**Results:**

We have developed a novel spike detection method for wheat plants involving, firstly, an improved colour index method for plant segmentation and, secondly, a neural network-based method using Laws texture energy for spike detection. The spike detection step was further improved by removing noise using an area and height threshold. The evaluation results showed an accuracy of over 80% in identification of spikes. In the proposed method we also measure the area of individual spikes as well as all spikes of individual plants under different experimental conditions. The correlation between the final average grain yield and spike area is also discussed in this paper.

**Conclusions:**

Our highly accurate yield trait phenotyping method for spike number counting and spike area estimation, is useful and reliable not only for grain yield estimation but also for detecting and quantifying subtle phenotypic variations arising from genetic or environmental differences.

## Background

Wheat is one of the three most important crop species worldwide with 700 million tonnes of grain produced annually [1]. However, with population growth, increasing demand and climate change threatening supply, greater effort is needed to ensure sustainable wheat crop production [2]. This translates into increased pressure on plant breeders to rapidly and accurately identify suitable wheat plant varieties that could be used for commercial production. In this effort, crop phenotyping by quantitative assessment of crop canopy features plays an important role as a quantifier of crop performance. It thus represents an important tool for identifying high-yielding novel varieties. One of the aims of digital crop phenotyping is to predict, non-destructively, the yield of a crop and preferably at an early stage in plant development.

The life span of cereal plants can be divided into four stages [3, 4] based on the Feekes scale: tillering, stem elongation, heading and ripening. Of the critical factors contributing to crop yield, tiller number is established at the early stage while spike number features in the mid-life of plant development. Other factors such as spike size, grain number per spike and grain weight feature at later stages. One aim of the phenotyping process is to understand plant development over time and its relevance to final yield. If, however, yield can be estimated at an early stage using early indicators alone, then the length of experiments can be reduced which would potentially accelerate breeding efforts; it would certainly reduce the cost per trial. However, to achieve this requires complex growth models that link early or middle plant development to final yield. Tillers are important initial components related to yield as they have the potential to develop grain-bearing spikes. However, the number of tillers a plant develops is not constant and will vary due to the interaction between genetic makeup, environmental conditions and agricultural practice. In this study, we focus on the heading growth stage and one of the yield measures—spike number, rather than tiller number. This will serve as the basis for a top-down approach to plant and growth modelling to be implemented later.

To date, there have been relatively few studies concerned with spike detection and specific characterisation [5–8]. Some spike characteristics, such as awn number, awn length and spike length were measured in wheat using morphological image processing of images taken of single spikes in order to classify the wheat variety in question [5, 6]. Lv [7] developed a spike identification method based on a back propagation neural network using Hu moments that measured seven characteristic parameters with images of individually cut spikes. A similar destructive spike measurement method was proposed by Hongju and Changing [8]. However, these methods are unsuitable for high-throughput, non-destructive, phenotyping for the purpose of identifying spikes from whole living wheat plants.

In this paper, we propose a novel approach for detecting spikes from digital images of wheat plants. We have observed that there is a difference in the texture features between spikes and leaves despite their colors being similar. This is particularly true at the early heading stage, where texture is defined as the spatial arrangement of color or intensity in a region of interest. Therefore, we propose to use Laws texture energies as texture features and use neural network for spike detection. The major advantage of our approach is that it is non-destructive and a high-throughput approach for spike detection which opens the door for phenotyping of spike traits in time sequences of plant images.

## Results and discussion

### Spike identification on living plants (single time point)

**Fig. 1:**
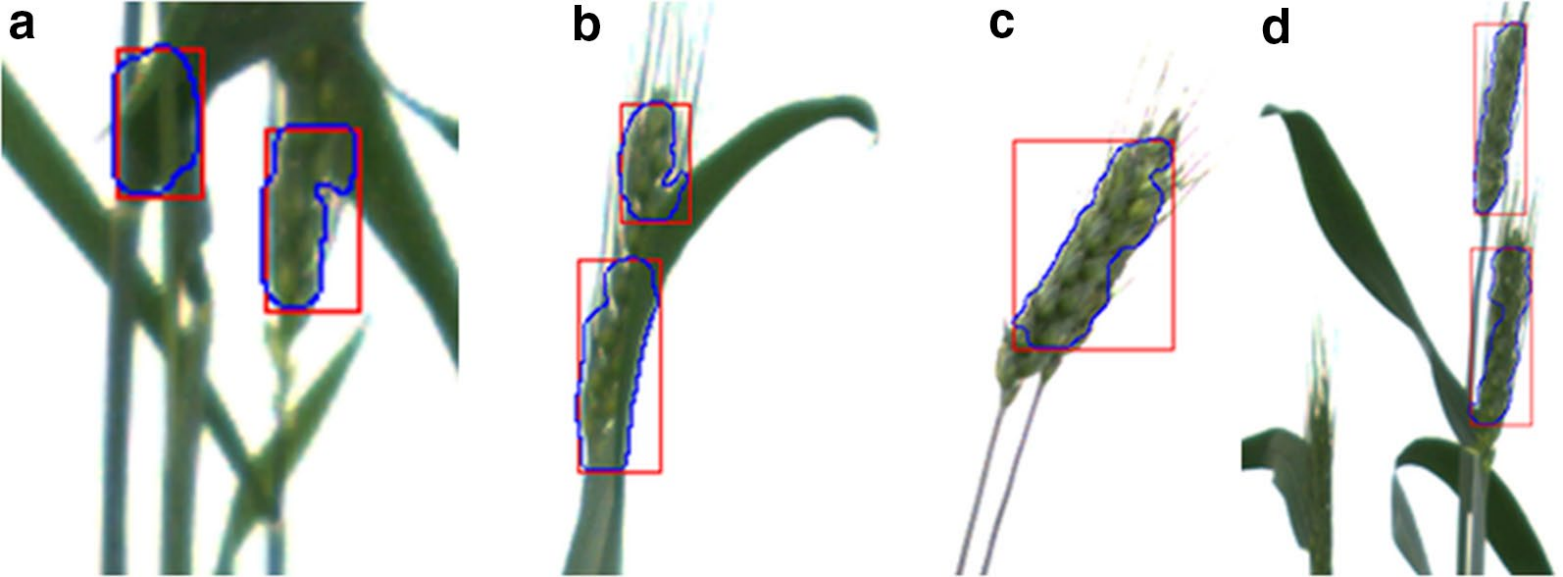
Examples of over counting (**a**, **b**) and undercounting(**c**, **d**) problems: **a** the misclassification of a leaf as a spike; **b** the miscount of one spike as two spikes; **c** the miscount of two overlapping spikes as a single spike; **d** a young spike not classified as a spike

**Fig. 2:**
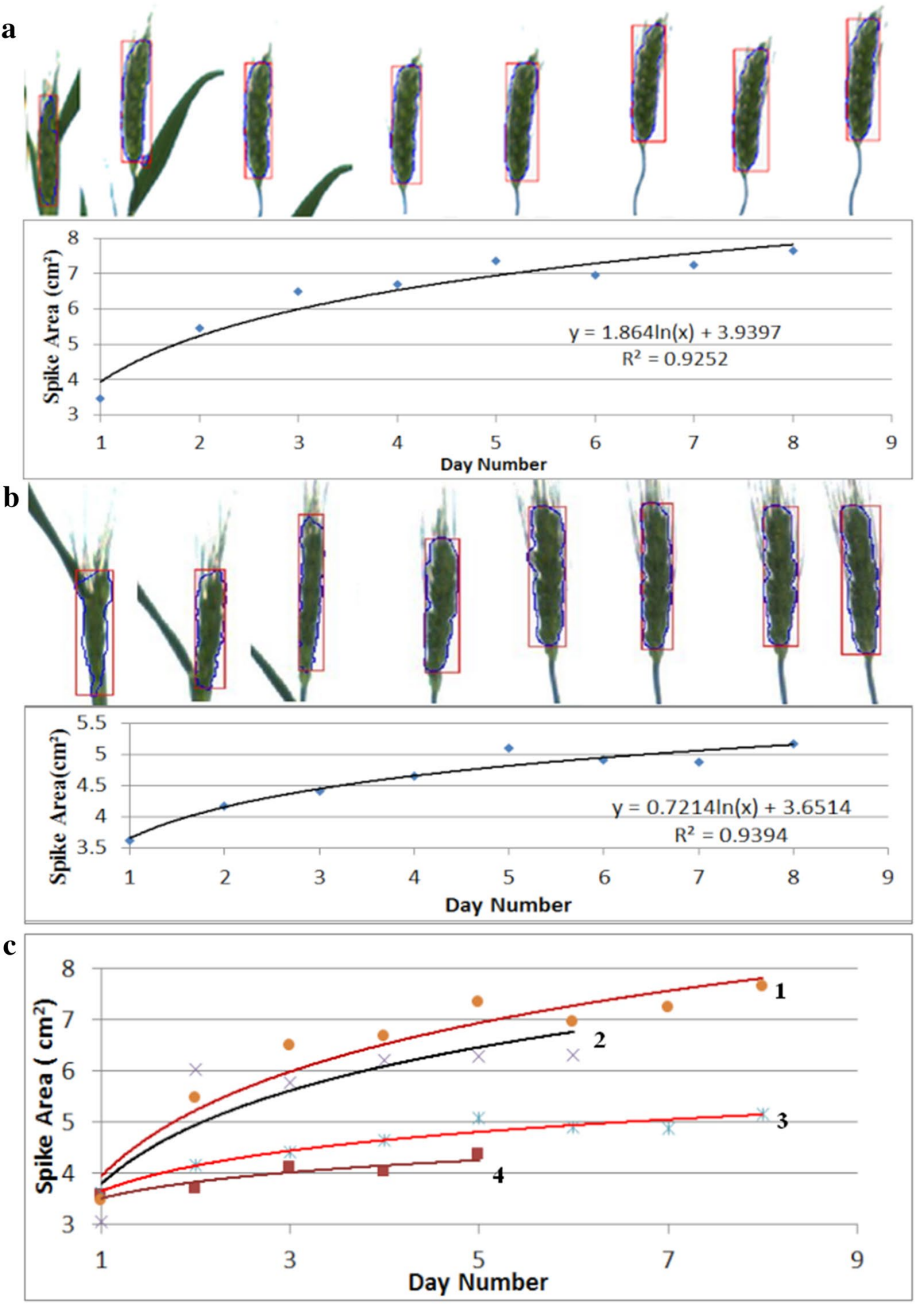
Example results from measuring the growth of individual spikes: **a**, **b** daily growth curves over a period of 8 days of two individual spikes with images of the detected spike on each day shown on the top of the curves, where day 1 is the day when the first spike became visible in the plant image and it is 42nd day after planting; **c** other examples of growth curves showed similar trends, which can be used to predict the growth trend in order to estimate yield at a much earlier stage. The end point is the last imaging day, while day 1 is the day when the spike became visible, it corresponds to the 42nd day after planting for curve 1, 3, the 44th and 45th day after planting for curve 2 and 4, respectively

We have argued that spike number is one key parameter contributing to the determination of yield of a cereal plant. Thus, even the seemingly simple task of counting the number of spikes is a valuable exercise. To validate the approach we have taken, analyses were conducted using images of 194 (the 2013 dataset) single wheat plants grown per pot. The images were taken from the tillering stage until about 1 week after the first spike emerged. The 194 plants were organised into six groups based on the number of spikes counted manually as shown in Table 1. By comparing the number of spikes detected using our automated method with manual counts, we find instances of over-counting and under-counting for each group. In any given image, detection errors arise usually due either to overcounting or undercounting. In the more complex case involving both an undetected spike and a falsely detected spike, a classification of over-counting or under-counting is appropriate depending on the area size of the miscounting. There are two main reasons for overestimating the number of spikes: (1) a misclassification of a leaf as a spike when the leaf and stem overlap (Fig. 1a), and (2) a miscount of one spike as two spikes (Fig. 1b). In any practical application, it is inevitable that there will be some classification errors. In our sample evaluation, some younger spikes were misclassified as two separated regions instead of a single spike (over-counting) as shown in Fig. 1b. In other cases, two overlapping spikes (red circled area of Fig. 1c) were classified as a single spike (undercounting). In some images, young spikes had not yet fully emerged from the sheath resulting in overlap with leaves, thus posing a considerable challenge to classification. Consequently, these young spikes were not classified as individual spikes (Fig. 1d). Generally, very young spikes do not share the same texture features as more mature spikes which leads to a miscounting error. However, our algorithm appears to work well for the majority of cases even at the early heading stage (see Fig. 1d), which is fortunate since one aim of the method is to quantify traits that contribute to predict final grain yield as early as possible.

**Table 1:** Results of counting the number of spikes

Number of spikes^a^	No of images^b^	Over-counting^c^	Under-counting^c^
0	41	5	0
1	57	5	0
2	44	2	3
3	45	4	5
4	5	0	1
5	2	0	1
Total	194	16	10

^a^The number of spikes per plant

^b^The number of images in the dataset which have the corresponding number of spikes based on manual counting

^c^The number of plants where spike number was over-counted or under- counted with the automated method compared to the manual count

We based the evaluation of our spike detection method on two sets of data (Table 2). The first dataset (2013) had a single wheat plant grown per pot. The second data set (2014) had either a single plant or four plants per pot. The resulting spike identification accuracy was 86.6 and 81.5%, respectively (Table 2). Not surprisingly, the more accurate result with the 2013 dataset, featuring only one plant per pot, was due to fewer instances of spike overlap with other spikes or with leaves appearing in images.

**Table 2:** Evaluation of spike detection

Experiment	Total number of images^a^	Correct^b^	Incorrect^b^	Accuracy^c^ (%)
2013	194	168	26	86.6
2014	206	168	38	81.5

^a^For 2013 dataset, we selected the image on the last imaging day for each plant; for 2014 dataset, in order to get more sample images, we took few images for each plant on the last few imaging days

^b^Manually check whether all the spikes in the image are detected, if all the spikes are recognized, and no any misclassified, we defined as correct; If there is some spikes are not recognized, or misclassified, we defined as incorrect

^c^ $$Accuracy = \frac{{N_{correct} }}{{N_{total} }} \times 100\%$$

We remark here that the evaluation thus far was based on individual images of plants at a single time point. However, using a time-series of images as well as images of the same plant from different perspective could improve the accuracy further.

### Measuring the growth of individual spikes

One other factor contributing to yield is spike size [9]. Consequently, in addition to detecting and counting the spikes, we also measured their projected area in the 2D digital images. Example results of measuring areas of individual spikes are shown in Fig. 2. This analyses was carried out on the 2013 data set. Results based on daily measurements of two plants over a period of 8 days are illustrated in Fig. 2a, b. It is clear that spikes grow faster in the first 4 or 5 days following their emergence, before asymptoting to their maximum size. However, the spike measured in Fig. 2b was partially enveloped by the leaf sheath on day 1 and this can result in either an overestimated or underestimated spike area. In Fig. 2a, b, we observe that there was no significant change in terms of the spike size during the last 4 days, when the plants were at the anthesis stage. It appears that the spike is close to its maximum size already within 5 days following emergence. This fact provides us with a means to an early estimation of final yield. More examples of growth curves for spikes are shown in Fig. 2c, with curves starting from the day of spike emergence and lasts to the end of the imaging period for this experiment. All plants had the same end date for imaging, but the date of spike emergence varied from plant to plant.

### Measuring the growth of spike area of whole plants (Mace in small pot)

**Fig. 3:**
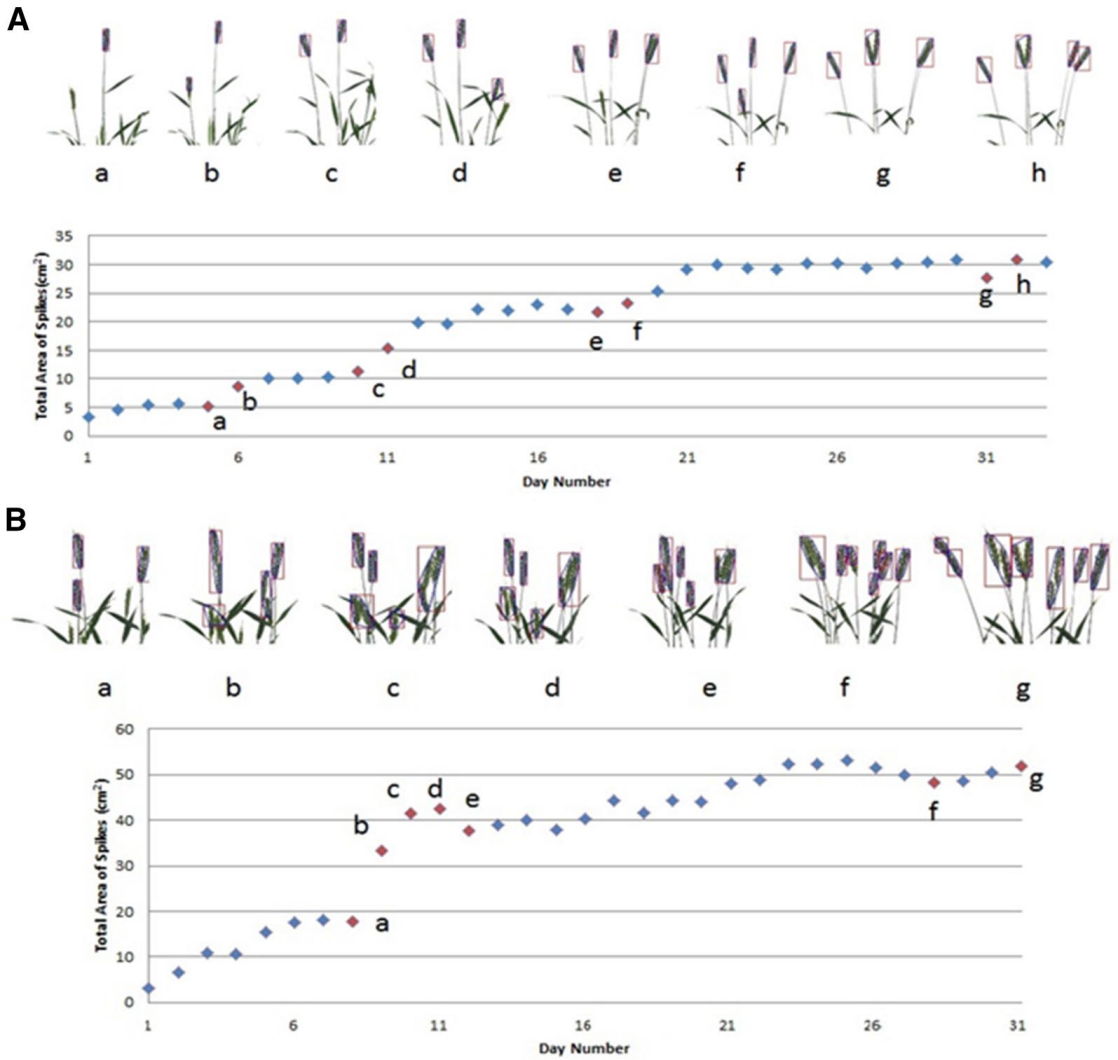
Example results from measuring the growth of whole plant: the daily growth within around a month of a single plant (cultivar Mace) in a pot for two different plants **A** and **B** was shown in the scatter chart, the end point is the last imaging day, and day 1 is the day when the first spike became visible in the plant image. The images *a*–*h* above the charts are the detected spikes corresponding to the points *a*–*h* in the scatter charts, which showed two main reasons for the bigger data changes in the charts: overlapping and new spikes came out

Next, we examined entire plants with multiple, rather than single, spikes using the 2014 dataset. The changes in spike areas of whole wheat plants over a period of about 1 month are illustrated in Fig. 3. In Fig. 3A, the total spike area increase in a step-wise manner. The images (a–h) in Fig. 3A exemplify the spike area estimation by our method and explain the step-wise increase. Although there is actually a continual increase in projected area as the plants develop, so long as spikes are still enclosed in their respective sheaths, even if only partially, our method does not identify or recognize them as spikes. Consequently the area of partially exposed spikes is not considered and does not contribute to the total area. However, once a spike is fully emerged it can be identified and its projected area is counted. This results in a stepwise increase in the total area. The addition of each new spike is highlighted in Fig. 3A by the jumps from “**a**” to “**b**” and “**c**” to “**d**”, or “**a**” to “**b**” in Fig. 3B. On the other hand, not all jumps are caused by the emergence of spikes from flag leaves. Two other complicating factors can result in step changes in spike area. For instance, there is the possibility of a spike changing its position from overlapping to non-overlapping state as illustrated by the estimates from “**g**” to “**h**” (Fig. 3A). On the other hand, the occasional step decrease in total area can be caused by one spike becoming occluded by another spike as seen in Fig. 3A-g and 3B-e. There are other complications too, such as the emergence of spikes and the overlapping of two spikes in the same image as illustrated by the estimates from “**e**” to “**f**” in Fig. 3B. In general, the main reasons for the significant increases in projected spike areas are the separation of overlapped spikes, the overlap of previously separated spikes and the emergence of new spikes.

### Measuring the growth of spikes under different nitrogen treatments

**Fig. 4:**
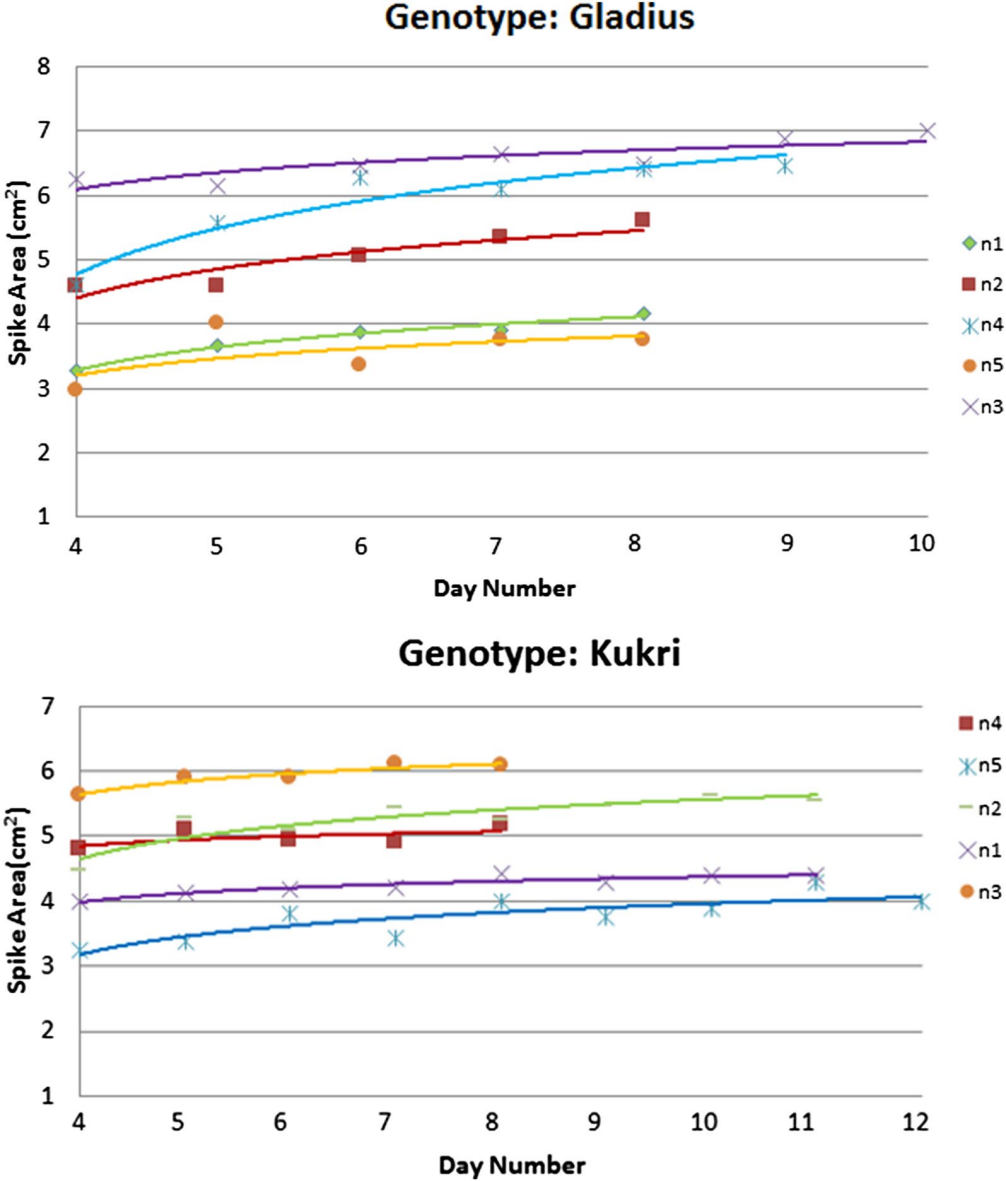
Example results from measuring spike growth under different nitrogen treatments: the measurement was carried on the first spike of plant for 2 genotypes (Gladius and Kukri) under 5 nitrogen treatments (n1, n2, n3, n4, n5). The end point is the last imaging day (49th day after planting), while the start point is the 4th day after the spike became visible in the plant image. Logarithmic model was applied for all the data set to get the curves

Spike emergence, development and growth are features that are dependent on both genotypic makeup and environmental (i.e., nutrient) condition. Consequently, we have investigated spike growth and development under a variety of stress conditions. In order to avoid the impact of spike overlap on our estimation of spike growth, this study focused only on the first spikes to emerge from individual plants. We studied plants from two wheat genotypes, each type subjected to five different nitrogen treatments (2013 dataset). Earlier tests have shown that spike area calculation can be inaccurate in the first few days after spike emergence, due to the presence of the envelope within the sheath as well as possible overlap with the flag leaf (Fig. 2). Therefore, the growth curves shown in Fig. 4 are referenced from the fourth day following initial spike detection and end on the last imaging day, day 49. For Gladius, the growth response corresponding to different treatments appeared to be parallel, and initially in the order of increasing nitrogen (n1–n3). However, further added (excess) nitrogen resulted in a progressively poorer growth response (n4–n5). We have not investigated the reason for the slightly more rapid growth rate of curve n4 compared with the responses to the other treatments. Generally, the biggest size was attained under the n3 treatment, while the smallest sizes resulted from the n1 and n5 treatments. It is also interesting to point out that the first spike came out 1 or 2 days earlier than under n3 treatment compared with other treatments. Thus, although the same reference point (4 days after first emergence) was used in the analysis, different absolute emergence days were observed. A similar qualitative response was found for the Kukri variety, although with some subtle differences: the spike emerged a few days earlier under n1, n2 (both on the 42nd day after planting) and n5 (41st day after planting) treatments, compared to n3 and n4 treatments (both on the 45th day after planting). It would seem from the above comparison that we can use the method to differentiate spike growth trends pertaining to different genotypes as well as subject to different stress treatments.

### Prediction of yield based on spike size

One of the principle aims of image-based phenotyping is to quantify plant traits non-destructively as a function of plant genotype, environmental conditions and time. However, it is also theoretically possible for image-based information derived at early stages of plant development to be utilised for the prediction of plant traits at later stages. In this study, we demonstrate this possibility by predicting final grain yield based on the spike size data obtained at the early heading stage.

There is, however, one major issue that needs to first be resolved for the purpose of making an absolute yield prediction. The issue relates to the spike number of a plant at the last imaging day being potentially different from the final number at day of harvest. To deal with this issue, we may focus only on predicting the average grain yield per spike, instead of the final total yield, using the size of the first spike of a plant for that analysis.

**Fig. 5:**
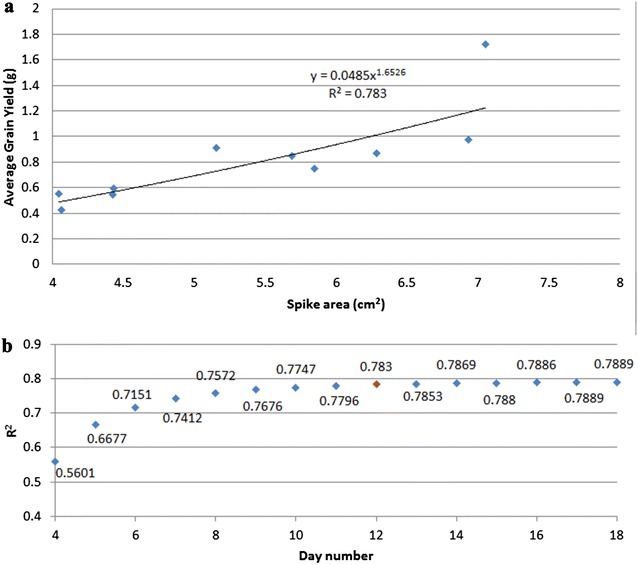
Relation between grain yield and spike area: **a** The horizontal axis is the area of single spike, and the vertical axis is the average harvest yield per spike. All the spike areas were predicted on the 12th day after spike became visible. **b** *R* ^*2*^ value of prediction based on the data of different day after spike visible

Although a better prediction of yield can be achieved using data from periods closer to the day of harvest, we shall use the information on spike growth patterns shown in Figs. 2 and 4 to estimate spike size well after the last imaging day. To this end we analysed the relation between the average final yield per spike and the area of the first spike, measured on day 12 after spike emergence (Fig. 5a). Using a power law function to model the data, we achieved a satisfactory correlation of *R* ^2^ = 0.783 between this model and our quantified spike size. It is worthwhile noting that the power law model improves in accuracy of representation (increased *R* ^2^ values) as more data from later periods is used. However, improvement beyond 10 days after first spike emergence is marginal. Taking time of plant growth and yield prediction accuracy into account, we recommend estimation of spike yield at day 10–12 after first spike emergence to achieve reasonable accuracy without the need for prolonged plant growth and imaging.

The power law model allowed us to predict grain yield per spike essentially at an early stage. It is also possible to estimate the grain yield potential of a whole plant using the combination of spike numbers and spike size. In the cases that were studied, we were actually able to predict yield using data as at the 8th day after the first spike became visible. We also find that the accuracy of the prediction can be further improved slightly if we measure for a longer period (i.e., up to 17 days after spike emergence).

## Conclusions

We present an effective method for cereal spike detection from digital images. We employ the neural network based method with Laws texture energy for spike detection [21]. The proposed approach has been evaluated on plant images achieving an accuracy higher than 80% in the identification of wheat spikes. We also demonstrated that the proposed method is able to determine both the number of spikes and the spike growth, which we indicate can be useful to quantify phenotypic traits for genetic variation and for treatment effects. Spike detection and final grain yield per spike were found to be highly correlated, providing the user with a potential algorithm with which to estimate final grain yield at earlier developmental stages. From an application prospective, the methodology can conceivably be used to estimate grain yield in the field. Consequently, it is possible to predict final grain yield at least 50–60 days prior to harvesting, which could provide growers with early decision making opportunities for additional practices. For example, if predicted yield is higher than originally designed, later unnecessary application of nitrogen fertilizer may be avoided (unless aimed to boost grain protein content). Finally, the proposed approach has the potential to be applied to other cereal crops such as barley and rice, and the concepts can also form the basis of a solid platform for non-cereal crops.

In future work, we shall explore the possibility of taking greater advantage of time-course image sequences of plants grown in individual pots to further improve the performance of spike detection method. A natural but ambitious extension we shall also consider is to adapt the algorithm to suit spike detection in field situations, which is arguably a more relevant enterprise addressing the need of plant breeders and cereal crop agriculture generally.

## Methods

### Plant material and growth condition

#### The 2013 dataset

Australian spring wheat cultivars Gladius and Kukri were grown in pots in glasshouse conditions between January and June, 2013. Preselected seeds of similar size were sown in pots filled with 2.5 kg of soil mix (coco-peat based potting media containing with different amount of N). Nitrogen was applied at sowing as urea at 10 mg (n1), 25 mg (n2), 75 mg (n3), 150 mg (n4), and 450 mg (n5) N/kg of soil. Four week old plants were phenotyped using the LemnaTec Scanalyzer 3D imaging system. RGB images were automatically captured daily for another 30 days. The plants were grown to maturity and harvested for their biomass and grain yield.

#### The 2014 dataset

Two common Australian bread wheat cultivars (Mace, Emu Rock) were grown in a coco-peat based potting mix containing slow release fertiliser (Osmocote). Multiple seeds were planted in 2.5 and 4.5 L draining pots and thinned out at the two-leaf stage to a single plant or four plants per pot, respectively. Seeds were planted on 20-12-2013 and grown and watered to bench capacity until 20-1-2014. Plants were then loaded onto The Plant Accelerator’s LemnaTec imaging system (LemnaTec GbmH, Aachen, Germany) for automated imaging and watering until 5-3-2014. Watering was maintained at 35% (w/w) gravimetric water content for the duration of the experiment. Average greenhouse temperatures were 25 °C during the day and 20 °C at night.

### Image capture

Visible light RGB images of wheat were taken daily using a LemnaTec 3D Scanalyzer (LemnaTec, GmbH, Aachen, Germany) at the Plant Accelerator^®^ (TPA), the University of Adelaide. At each time point three images were taken, two side view images at 90° horizontal rotation and a top view image. RGB images were taken with a Basler Pilot GigE Vision Camera (piA2400-12 gm/gc) with a 2454 × 2056 resolution and stored in PNG format. Since the side view images provided more information compared to the top view images, we only used side view images in this study.

### Image processing pipeline for spike detection

**Fig. 6:**
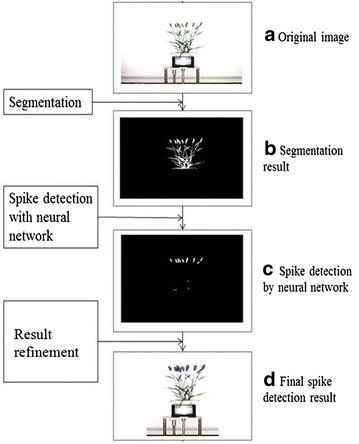
Image processing pipeline for spike detection. **a** orignial image; **b** the segmented plant image; **c** the initial detected spikes; and **d** the final detected spikes labelled in the original image

The flow chart in Fig. 6 shows the image processing steps used for spike detection, with image examples from our experiments. We developed our spike detection algorithm within the Matlab environment. In order to extract visual characteristics or features of plants from images, plant regions needed to be separated from the background by a segmentation process. For this purpose we used a color index-based method. A morphology algorithm was applied to binary images which were segmented using color indices to remove unwanted background pixels. An example of the segmentation result is shown in Fig. 6b after foreground (plant) segmentation and spikes were detected in the segmented plant images. In all cases spikes emerged from the top of the plant, as observed from the side-view TPA images. Therefore, to separate spikes from leaves, we applied a neural network-based Laws texture energy method to distinguish spikes from leaves (a straightforward height threshold analysis was not always successful as spikes could grow at different heights). An example result of spike detection using the neural network scheme is shown in Fig. 6c. The residual noise in the result was removed using a morphology algorithm based on height and area thresholds.

Two of the important objectives of this study were to detect the emergence of spikes and to count the number of spikes present. To avoid the problem of single spikes appearing as separate regions, the morphological closing (the dilation and then erosion) algorithm was used to integrate regions belonging to the same head into one region. To obtain the correct number of detected spikes it is necessary to resolve any issue arising from spike overlap (with leaves or other spikes). As individual spikes generally have similar size and shape, as shown in Fig. 6, we used the average size as a criterion to detect spike overlap. Two geometric parameters, the average area and perimeter, were used for this task. If the size of a detected region is deemed too large, relative to the average spike size, it is classified as overlapped spikes and treated as two spikes. In this way, we can reduce spike counting error from the initial counting. An example of the final result is shown in Fig. 6d.

### Image segmentation using colour indices

Colour indices are widely used for plant segmentation from image backgrounds [10–14]. Five colour indices: *r* − *g*, *g* − *b* (*g* − *b)/(r* − *g)*, and *2* *g* − *r* − *b,* derived using chromatic coordinates (r, g and b for red, green, and blue) and modified hue, were tested to distinguish living plant material from background [5]. By trial and error, it was established that the best segmentation result could be achieved using modified hue and the *2g* – *r* − *b* (excessive green: *ExG*) contrast index. In addition, an improved colour index, Excess Green minus Excess Red (*ExG* − *ExR*) [11], where *ExR* = *1.4r* − *g*, was compared to the more commonly used Excess Green (*ExG*). The excessive green index relies on a calculated threshold value to convert the index near-binary to a full-binary image, while the improved index (*ExG* − *ExR*) does not require a special threshold calculation. Plant pixel values become positive, while all remaining background pixels become negative. The index is capable of self-generating a binary image with a constant threshold of zero. Thus, the improved index (*ExG* − *ExR*) was used in this study, which is given by [11]:

1 $$ExG - ExR = 3g - 2.4r - b.$$

### Spike detection with neural network based Laws texture energy method

Texture analysis is an important specific methodology in computer image analysis for classification, detection and segmentation. Some of the most popular texture feature extraction methods are based on grey level co-occurrence statistics [15, 16], wavelet packets approaches [17], filtering methods like morphological filters, Fourier filters, random field models [18], Gabor filters [19] and local binary patterns [20]. Each method offers certain advantages and some disadvantages in discriminating texture characteristics. The method of choice depends on the problem at hand.

Laws texture energy method [21] has been used for many applications [17, 22–24]. This method seeks to classify each pixel of an image by mapping each pixel onto a texture energy plane. The mapping process is fast, requiring only convolutions and simple moving window techniques. This method uses micro-texture and macro-texture measures. Micro-texture measures are computed within very small overlapping windows. The windows are typically 3 × 3 or 5 × 5, small enough to make it unlikely that more than a single texture region exists within the window. Macro-texture measures are large-window summaries of the micro-features. Macro-windows must be large enough to include a representative sample of the image texture. A micro-window is used to measure the gray irregularities within a small region to form properties, while the macro window is used to find the statistics of properties in a larger window (normally mean or standard deviation). Laws method is simple but effective and can handle changes in luminance, contrast, and rotation without histogram equalization or other pre-processing operations. In this study, we used Laws local texture energy measures as features for the spike detection, typical 5 × 5 was chosen as micro-window size, and 25 × 25 as macro-window size. Laws developed a set of two-dimensional convolution masks typically used for texture discrimination that are derived from simple one-dimensional convolution masks. Equation (2) shows the set of one-dimensional convolution masks of length three. Each of these one-dimensional masks is associated with an underlying microstructure which is the level detection, edge detection and spot detection.

2 $$\begin{aligned} {\text{L}}3 = \left[ {\begin{array}{*{20}c} 1 & 2 & 1 \\ \end{array} } \right] - {\text{Level}}\,{\text{Detection}} \hfill \\ {\text{E}}3 = \left[ {\begin{array}{*{20}c} { - 1} & 0 & 1 \\ \end{array} } \right] - {\text{Edge}}\,{\text{Detection}} \hfill \\ {\text{S}}3 = \left[ {\begin{array}{*{20}c} { - 1} & 2 & { - 1} \\ \end{array} } \right] - {\text{Spot}}\,{\text{Detection}} \hfill \\ \end{aligned}$$

From these one-dimensional convolution masks, 9 different two-dimensional convolution masks can be generated by convolving a vertical one-dimensional mask with a horizontal one-dimensional mask. As an example, the L3E3 mask is found by convolving a vertical L3 mask with a horizontal E3 mask. 8 of the 9 two-dimensional convolution masks are zero-sum except the L3L3 mask. A listing of all 3 × 3 mask names with zero-sum is given below:

3 $$\begin{aligned} {\text{L3E3}} = {\text{L3}}^{\prime}*\text{E3}; \quad {\text{E3S3}} = \text{E3}^{\prime}*\text{E3}; \hfill \\ {\text{L3S3}} = \text{L3}^{\prime}*\text{S3}; \quad {\text{S3L3}} = {\text{S}}3^{\prime}*\text{L3}; \hfill \\ {\text{E3L3}} = \text{E3}^{\prime}*\text{L3}; \quad {\text{S3E3}} = \text{S3}^{\prime}*\text{E3}; \hfill \\ {\text{E3E3}} = \text{E3}^{\prime}*\text{E3}; \quad {\text{S3S3}} = \text{S3}^{\prime}*\text{S3}; \hfill \\ \end{aligned}$$

To build up a set of texture energy measures for each pixel in a digital image, two steps were performed [17]. The first step involves convolving the whole image by zero-sum masks. The 2D convolution of the image ***I*** and mask ***A*** of size (2a +1) by (2a + 1) is given by the relation:

4 $$\begin{aligned} F(i,j) & = (A*I)(i,j) \\ & = \sum\limits_{p = - a}^{a} {\sum\limits_{l = - a}^{a} {A(p,l)} } I(i + p,j + l) \\ \end{aligned}$$

where (2a + 1) is the micro-window size * denotes 2D convolution and the mask A can be one of masks in (3).

The second step consists of a windowing operation, which is done by looking in a local neighborhood (macro-window of size (2n + 1) × (2n + 1)) and calculating the mean deviation around each pixel according to:

5 $$E(i,j) = \frac{1}{{(2n + 1)^{2} }}\sum\limits_{p = i - n}^{i + n} {\sum\limits_{l = j - n}^{j + n} {\left| {F(p,l)} \right|} }$$

where *E*(*i*,*j*) is the so called texture energy measure (Laws), which is used in this study for the classification process.

**Fig. 7:**
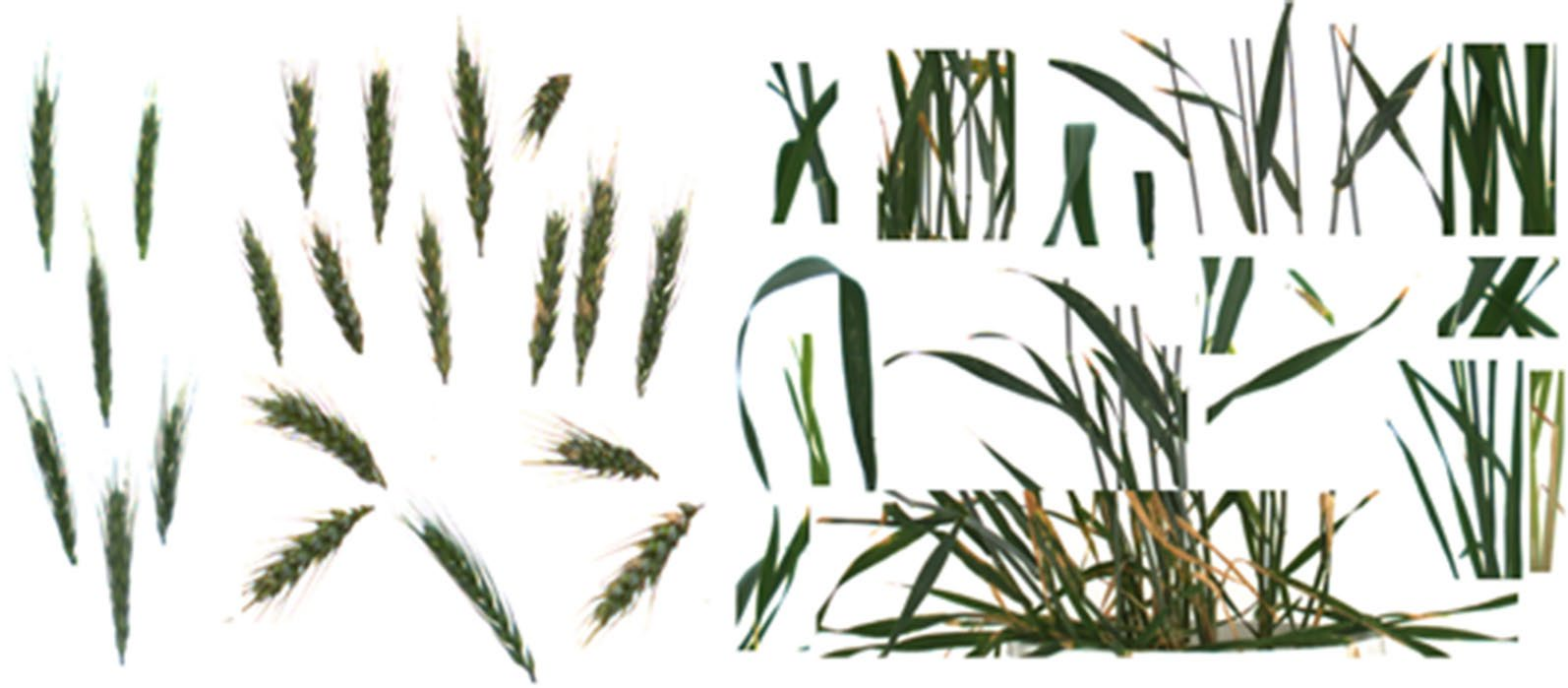
Sample for neural network trainings: spike samples (left) and leaf samples (right)

**Fig. 8:**
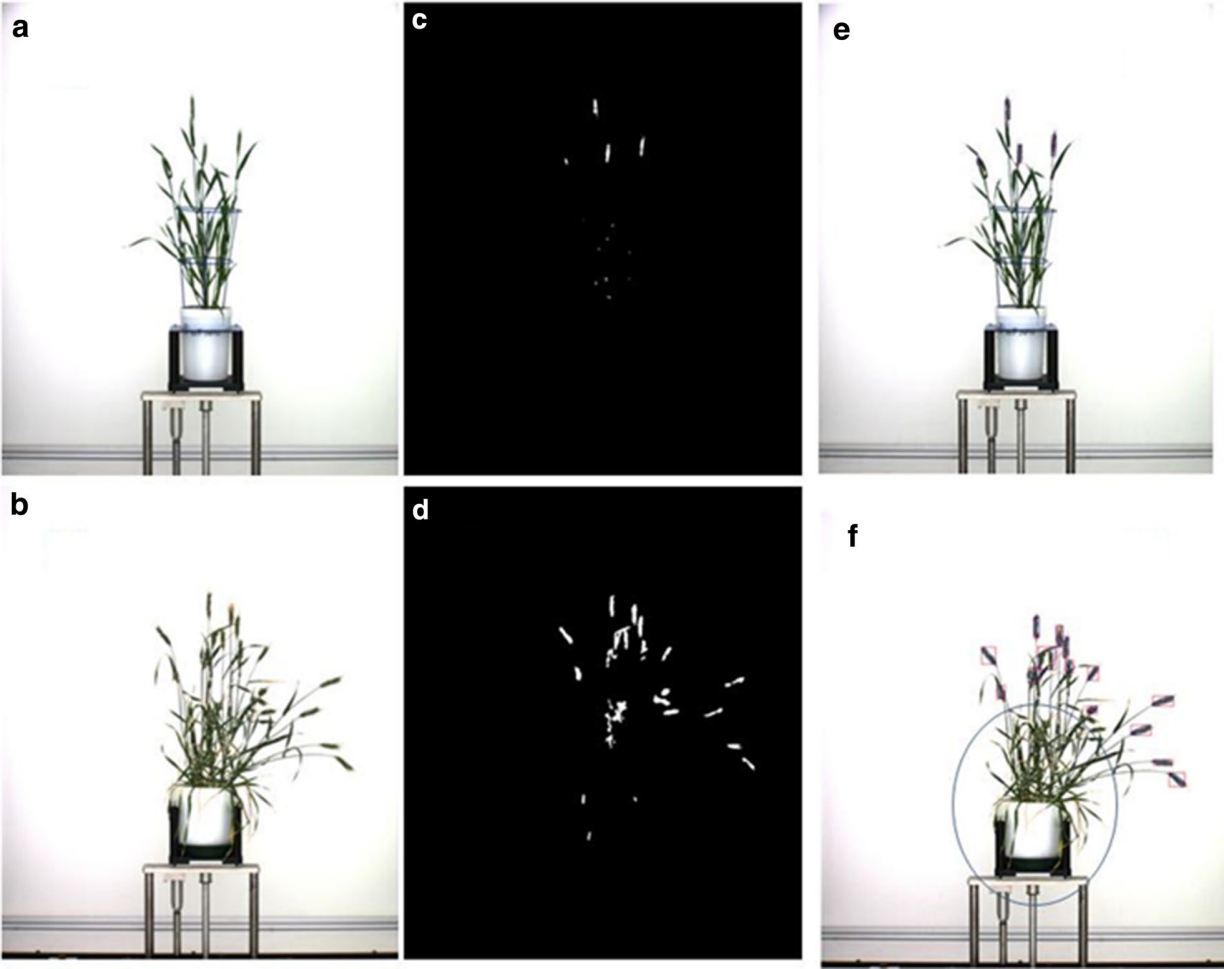
Initial pixels based classification and noise removing: **a**, **b** original image; **c**, **d** the initial pixels based spike identification of **a**, **b** respectively; noise removed by taking all the pixels above the blue frame for 2013 dataset (**e**), and noise removed by taking all the pixels outside the blue circle for 2014 dataset (**f**)

A neural network was used to perform the classification task in this study, which has one hidden layer, and 10 neurons in the layer. 21 image patches from Dataset 2013 and 2014 were manually selected to obtain the sample of spikes, while 14 image patches for the sample of leaves, considering the variation of the growth stages and the positions (as shown in Fig. 7). The total number of samples (pixels) of spike and leaf are 19,041 and 76,154, respectively, where around 15% of total samples are taken for both validation and testing (as shown in Table 3) and remainder are used for training. Note, images used for this experiment were excluded from the spike counting experiment. For each pixel, the inputs are the eight texture features mentioned above, and the output is two classes: leaf or spike. The accuracy of the classifier calculated from the confusion matrix is 92.3%. Figure 8c, d shows two examples of the initial pixels based classification.

**Table 3:** Accuracy of the classification

	Training	Testing	Validation	Total
Spike samples (pixels)	13,372	2890	2779	19,041
Leaf samples (pixels)	53,265	11,389	11,500	76,154
TP rate^a^ (%)	80.2	79	78.8	79.9
TN rate^a^ (%)	95.7	95.6	95.9	95.7
Accuracy^a^(%)	92.5	92.3	92.4	92.4

^a^Accuracy, TP rate and TN rate were defined as follows:

$$Accuracy = \frac{TP + TN}{TP + FP + TN + FN}$$; $$Tprate = \frac{TP}{TP + FN}$$; $$TNrate = \frac{TN}{FP + TN}$$

where *TP, TN, FP*, and *FN* represent the numbers of true positives, true negatives, false positives, and false negatives, respectively

### Spike detection result refinement

#### Noise removing

As shown in Fig. 8, for dataset 2013, as spikes grow above the frame in the image, we simply extracted the region above the blue frame in order to remove the noise (Fig. 8e). But for dataset 2014, blue frames were not used in the experiment. In order to remove most noise on the bottom, we defined a circle centred at the centre of the top line of the pot, and the radius was calculated as 60% of the total height of the plant (Fig. 8f). Pixels within the circle will be classified as non-spike pixels.

**Fig. 9:**
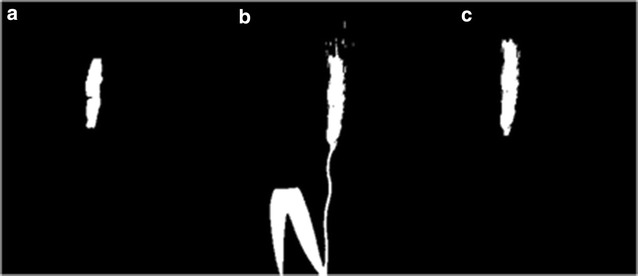
Spike area calculation: **a** the spike area detected by neural network; **b** the spike area in the segmented image; **c** the final spike area used to calculate the pixel area in this research

 As not all the pixels of a spike can be detected by our proposed method (as shown in Fig. 8c, d. If we use the detected area of a spike to estimate the spike size, we would underestimate the real size of the spike due to the edge effects of texture features. So it’s necessary to further refine the spike detection. In order to accurately estimate the sizes of all the spikes, we used the spike area in segmentation image in our research. The method is to find the spike area in segmentation image corresponding to the detected spike region by first doing a logical ‘and’ operation for each detected spike region and all the regions in segmentation image, then the region in segmentation image was extracted if the ‘and’ operation result is true. Figure 9a shows an example of a spike detected by our proposed method, the corresponding area in segmentation image is shown in Fig. 9b, and Fig. 9c is the final result of the spike area used in our research.
